# Give me my pudding: a case report of hoarding symptoms in a first psychotic episode of a woman with schizotypal personality

**DOI:** 10.1192/j.eurpsy.2025.1905

**Published:** 2025-08-26

**Authors:** M. Almada, T. Pessoa, A. Miguel, R. Correia

**Affiliations:** 1 Psychiatry, ULSGE, Vila Nova de Gaia, Portugal

## Abstract

**Introduction:**

Schizotypal personality disorder is characterized by social and interpersonal deficits, eccentric behaviour, unusual beliefs, magical thinking and blunted affect, and it may represent a predisposition for psychosis. Hoarding disorder, which has recently been recognised in ICD-11, is defined by accumulation and persistent difficulty in discarding ordinary possessions, leading to interfering levels of clutter that compromise the use or safety of living spaces. Hoarding behaviour can occur in a variety of neuropsychiatric disorders, including psychotic disorders. Research suggests an association between schizotypal traits and hoarding symptoms including cognitive deficits, emotional dysregulation or impaired insight, which are also characteristic of psychosis.

**Objectives:**

We aim to present a case of hoarding disorder in a woman with schizotypal personality traits which presented with a first psychotic episode at her fifties.

**Methods:**

We describe a case report and a non-systematic review on the subject.

**Results:**

A 55-years-old woman was admitted to psychiatric emergency department with behaviour changes, marked by significant neglect in self and health-care in addition to social and emotional isolation. She also exhibited somatic, persecutory and autoreferential delusions, as well as auditory hallucinations. She believed that her body had no production of saliva and her meals mostly consisted of chocolate pudding, since she was convinced this was the only food her organism could tolerate. As a consequence, her nutrition was fairly neglected and severe potassium deficits were observed with electrocardiogram changes and fainting episode. Beyond these symptoms, a severe hoarding pattern was found which had worsened over the last years. Supported by family meetings and psychological personality assessment, dysfunctional personality traits stood out, with a difficulty in interpersonal relationships and adaptation to external reality. During hospitalization, we found that psychotic symptoms had months of evolution. Antipsychotic medication was initiated (Paliperidone) with significant improvement of symptomatology, but not full remission.

**Conclusions:**

Upon a comprehensive assessment, we concluded that the patient’s dysfunctionality was mainly due to schizotypal personality disorder rather than the acute psychotic episode. This case also suggests the importance of assessing personality traits, in particular schizotypal, in patients with hoarding symptoms. An overlap between hoarding symptoms and schizotypy has previously been reported in literature. Therefore, we highlight that distinction among schizotypal personality traits, psychotic and hoarding symptoms can be challenging among clinical practice. In overall, a broad assessment of symptoms is warranted in order to better understand what is in the basis of patient’s dysfunctionality and ensure and effective treatment.

**Disclosure of Interest:**

None Declared

